# Particle Beam Radiation Therapy for Skull Base Sarcomas

**DOI:** 10.3389/fonc.2020.01368

**Published:** 2020-09-16

**Authors:** Jing Yang, Weixu Hu, Xiyin Guan, Jiyi Hu, Jing Gao, Xianxin Qiu, Qingting Huang, Wenna Zhang, Lin Kong, Jiade J. Lu

**Affiliations:** ^1^Department of Radiation Oncology, Shanghai Proton and Heavy Ion Center, Shanghai, China; ^2^Shanghai Engineering Research Center of Proton and Heavy Ion Radiation Therapy, Shanghai, China; ^3^Department of Radiation Oncology, Shanghai Proton and Heavy Ion Center, Fudan University Shanghai Cancer Hospital, Shanghai, China

**Keywords:** proton beam radiation therapy, carbon-ion beam radiation therapy, sarcoma, skull base, charged particle radiation therapy

## Abstract

**Background:** To report the clinical experience of carbon-ion and proton radiation therapy for skull base sarcomas.

**Methods:** An analysis of the retrospective data registry from the Shanghai Proton and Heavy Ion Center for patients with skull base sarcomas was conducted. The 1-/2-year local relapse-free, distant metastasis-free, progression-free, and overall survival (LRFS, DMFS, PFS, OS) rates as well as associated prognostic indicators were analyzed. Radiotherapy-induced acute and late toxicities were summarized.

**Results:** Between 7/2014 and 5/2019, 62 patients with skull base sarcomas of various subtypes received carbon-ion radiation therapy (53), proton radiation therapy (5), or proton radiation therapy + carbon-ion boost (4). With a median follow-up of 20.4 (range 2.73–91.67) months, the 1-/2-year OS, LRFS, DMFS, and PFS rates were 91.2%/80.2%, 89.2%/80.2%, 86.0%/81.1%, and 75.8%/62.9%, respectively. Grade 3 mucositis and grade 4 hemorrhage were observed in 1 patient for each. Only grade 1 and grade 2 toxicities were observed except for the same patient with grade 4 acute toxicity died of severe hemorrhage (grade 5). Multivariate analyses revealed the lack of prior RT was an independent favorable prognostic factor for OS, PFS, and LRFS, age under 40 was associated with improved OS, early T-disease (T1/2) showed a significant association with better PFS.

**Conclusion:** With few observed acute and late toxicities, particle beam radiation therapy provided effective tumor control and overall survival for patients with skull base sarcomas.

## Introduction

Bone and soft-tissue sarcomas of the base of the skull (SBS) are rare and account for < 1% of all head and neck malignancies (1–5). Surgery is the treatment of choice for SBS regardless of the histology subtypes. However, *en bloc* resection with sufficient surgical margin of skull base tumors is universally challenging due to the complexity of the anatomy. Although adjuvant radiation is commonly recommended, both the anatomical complexity, and radioresistant nature of most histology subtypes of SBS negated the efficacy of photon-based radiation, including the more conformal intensity-modulated radiotherapy (IMRT) technology. As such, despite aggressive multidisciplinary approaches, the prognosis of patients with SBS is poor as compared with patients with sarcomas in other anatomical regions (6, 7). For SBS patients with unresectable or inoperable disease, prognosis is usually dismal after radiotherapy (8–12).

Accelerated charged particle (e.g., protons, helium, and carbon ion) beams deposit relatively low energy in the path of traveling in the body but distribute most dose just before they stop at the Bragg peak. Such physical feature of particle beams makes it possible to deliver high-dose radiation to the tumor while limiting the dose to the organs at risk (OARs) close to the tumor. Also, heavy-ion (such as carbon ion) beams have higher linear energy transfer and greater relative biological effectiveness (RBE), which range between 2 and 5, depending on the beam energy, tissue and cell types, and fraction dose as compared with photon/proton (13, 14). Both features are critical for the treatment of radioresistant tumors that occur near-critical or sensitive OARs such as SBS. However, evidence supporting the use of particle beam radiation therapy (PBRT) for the management of SBS is scant. This paper reports the clinical results, in terms of disease control, survival, and treatment-associated adverse effects, of a relatively large group of SBS patients treated with PBRT at the Shanghai Proton and Heavy Ion Center (SPHIC) over the past 5 years.

## Methods

This is a retrospective study and was approved by the Institutional Review Board of SPHIC.

### Patient Population and Pretreatment Workups

Due to the significant difference of the biological behavior as compared with other histological subtypes of SBS, patients with chordoma of the skull base or cervical spine were not included in this analysis. After this exclusion, a total of 62 consecutive and non-selected patients with skull base bone and soft-tissue sarcoma who received PBRT with definitive intention at the SPHIC between July 2014, when the first SBS patient was treated at SPHIC, and May 2019 were included in this retrospective analysis.

All patients were evaluated according to the standardized pre-radiation workups, including a complete history, and physical examination, imaging studies (contrast-enhanced MRI preferred, but CT allowed if MRI is contraindicated) of the head and neck region, routing lab tests (complete blood count, serum electrolytes, and renal/hepatic function tests), fluorodeoxyglucose positron emission tomography/CT (or thoracic/abdominal CT and bone scan), and EKG. All patients were staged according to the American Joint Committee on Cancer, seventh edition, tumor–nodes–metastases staging system regardless of the time of diagnosis, as differentiation (grades) were not available to some patients diagnosed after January 2018.

### Particle Beam Radiation Therapy

All patients were registered and immobilized using AlphaCradle® and customized thermoplastic masks in the supine position. CT scans for PBRT planning were taken at 1.5-mm slice thickness without contrast from the vertex to the inferior margin of clavicular heads. The fusion of MRI taken in treatment position with immobilization mask and planning CT was applied for all patients before target volume delineation.

The gross tumor volume (GTV) was defined as the gross tumor visualized on imaging studies or clinical examination. GTV with a 1–3-mm expansion was treated to the prescribed dose to the tumor. CTV for gross and subclinical disease included an area with risk for subclinical disease as well as the pretreatment tumor bed for patients who received surgery and/or chemotherapy before PBRT. A maximum of a 5-mm margin was typically added to the CTV for the planning target volume (PTV) to mitigate uncertainty about dose distribution and potential setup errors.

**Figure 1:**
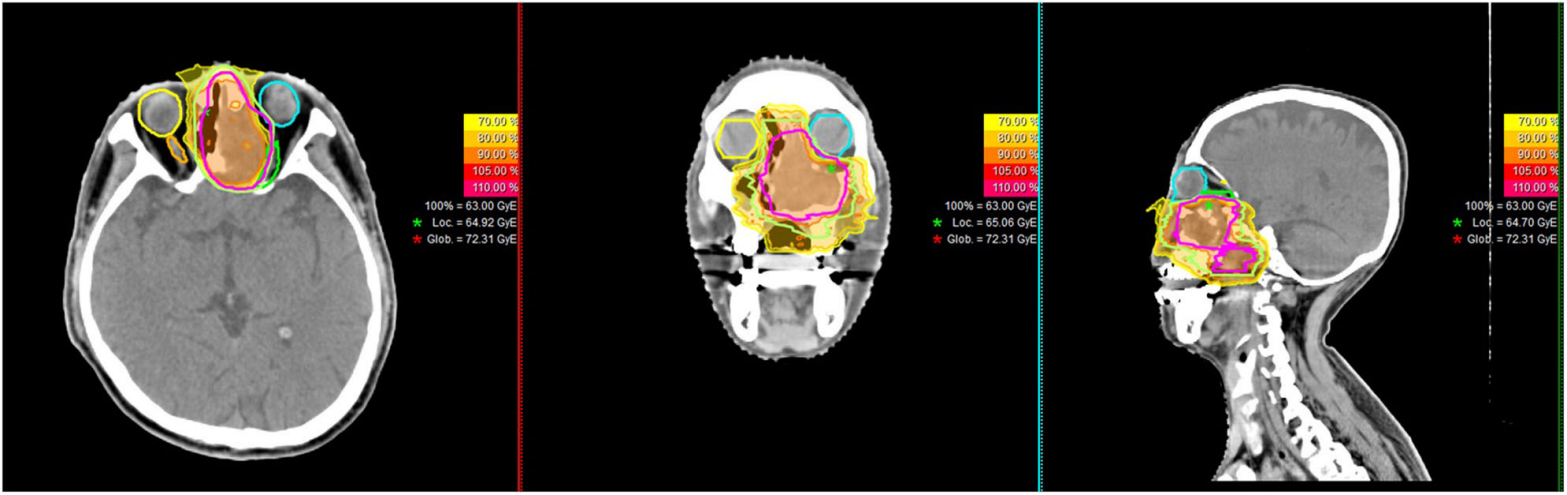
A typical treatment plan of a patient with soft-tissue sarcoma of the skull base.

PBRT (both proton and carbon-ion therapies [CIRT]) were delivered with pencil beam scanning (PBS) technology, typically consisted of beams from two to three directions. PBRT planning was performed using the Syngo® treatment planning system (version VC11 and 13) (Siemens, Erlangen, Germany). Individual factors such as patient positioning reproducibility and/or beam angles were chosen for optimal dosimetry. At SPHIC, only multi-field optimization was used for planning of PBRT using PBS technology for head and neck cancer patients. A typical treatment plan is displayed in Figure 1. The range uncertainty of our IONTRIS particle treatment system is ± 3.5% but could be modified according to the dose constrains of adjacent OARs. Setup accuracy was confirmed daily with orthogonal X-ray using bony landmarks as reference.

Doses of PBRT were measured by Gy (RBE) to account for the RBE differences as compared with photon-based RT. The dose constraints of the OARs were based on the TD5/5 described by Emami et al. (15) except for the optic nerves (D_20_ < 30 Gy [RBE]), brain stem (D_max_ < 45 Gy [RBE]), spinal cord (D_max_ < 30 Gy [RBE]), and temporal lobes (V_40_ < 7.66 cc; V_50_ < 4.66 cc), which were set using the previous experience from the National Institute of Radiation and Quantum Science of Japan (16). For patients who received salvage re-irradiation, the previous RT plans were obtained, and the doses to the OARs were identified. Recovery from the previous radiation therapy dose was set at 70% regardless of the latent time between the two courses of RT (17).

### Systemic Therapy

Chemotherapy and targeted therapy were administered at the discretion of the referring medical oncologists. Due to the substantial differences in the biological behaviors of different histologic subtypes of SBS, various regimens and schedules of systemic therapy were used. However, no patient with chondrosarcoma received systemic therapy. Concurrent systemic therapy was allowed during PBRT.

### Follow-Up and Toxicity Evaluation

Patients were evaluated weekly during PBRT for acute toxicities, response to treatment, and the potential need for replanning for PBRT due to substantial anatomical alteration. Weekly verification CT scans were typically performed after the second week of PBRT to assess any changes in anatomy. After the completion of PBRT, all patients were required to be followed up based on the standardized institutional follow-up protocol. The first follow-up was set at 4 weeks post-treatment; then, patients were examined every 3 months for the first 2 years, every 6 months up to the fifth year, then annually after that. Non-local or domestic patients unable to follow up in person were followed locally, and results were communicated. The Common Terminology Criteria for Adverse Events (CTC.AE) version 4.03 was used to grade acute (from the start to up to 3 months after the end of PBRT) as well as late adverse effects that occur any time after 3 months after PBRT until last follow-up for this group of patients.

### Statistics

The progression-free survival (PFS), locoregional relapse-free survival (LRFS), and distant metastasis-free survival (DMFS) rates were calculated from the start of PBRT, and the overall survival (OS) rates were from the diagnosis using the Kaplan–Meier method. Univariate and multivariate analyses on survivals were performed using the Kaplan–Meier method (with log-rank test) and Cox proportional hazards model. *P* < 0.05 were considered statistically significant. All statistical analyses were performed using SPSS (version 18.0).

## Results

### Study Population

The diagnosis of all 62 patients with skull base bone or soft-tissue sarcomas (chordoma excluded) were confirmed histologically. No patient presented with distant metastasis at diagnosis. Twenty-eight (18) patients presented with soft-tissue sarcoma, 28 presented with chondrosarcomas, and 6 with osteosarcomas. Thirty-three patients were diagnosed with T3/4 disease and accounted for 53.3% of the cohort. Only 1 patient presented with N1 disease. Seventeen cases had previous photon-based radiation therapy and received salvage CIRT. Among these 17 cases, 12 patients suffered radiation-induced second primary sarcomas. The characteristics of patients and their disease are detailed in Table 1.

**Table 1:** Characteristics of the 62 patients, their disease, and treatments.

**Characteristic**	**No**.	**%**
Sex		
Male	37	59.7
Female	25	40.3
Age (years)		
Median	38	
Range	14–71	
Histology		
Chondrosarcoma	28	45.2
Rhabdomyosarcoma	8	12.9
Spindle cell sarcoma	6	9.7
Osteosarcoma	6	9.7
Pleomorphic sarcoma	3	4.8
Others 1–2 of each	11	17.7
T-classification		
1	28	45.2
2	1	1.6
3	5	8.1
4	28	45.2
N-classification		
0	61	98.4
1	1	1.6
2	0	0.0
3	0	0.0
Re-irradiation		
Yes	17	27.4
No	45	72.6
Second primary		
Yes	12	19.4
No	50	80.6
Surgery		
R0 + R1	15	24.2
R2 + biopsy + no surgery	47	75.8
Chemotherapy		
Yes	17	27.4
No	45	72.6
PBRT types		
PRT	5	8.1
CIRT	53	85.5
PRT + CIRT	4	6.5
GTV (cm^3^)		
Median	47.45	
Range	0–1003.97	

*PRT, proton radiotherapy; CIRT, carbon-ion radiotherapy*.

### Particle Beam Radiation Therapy

All patients received PBRT according to the planned schedule. No patients had an unplanned treatment break. Four patients (three with chondrosarcoma and one with rhabdomyosarcoma) treated at the beginning of our clinical services received proton therapy followed by a CIRT boost. Five patients with chondrosarcoma accrued to our phase II randomized trial (proton vs. carbon ion for chondrosarcoma) received intensity-modulated proton therapy only to 64–70 Gy (RBE)/32–35 Fx depending on the dose constraints of the OARs. Fifty-three patients received intensity-modulated CIRT regimens using our dose escalation [54–73.5 Gy [RBE]/18–23 Fxn], randomized trials [70 Gy [RBE] in 20 fractions for chondrosarcomas only], or standard institutional protocol [CIRT to 63 Gy [RBE]/18 fractions to 70 Gy [RBE]/20 fractions depending on the pathology types, tumor volumes, and dose constrains of the OARs].

### Disease Control and Patients' Survival

**Figure 2:**
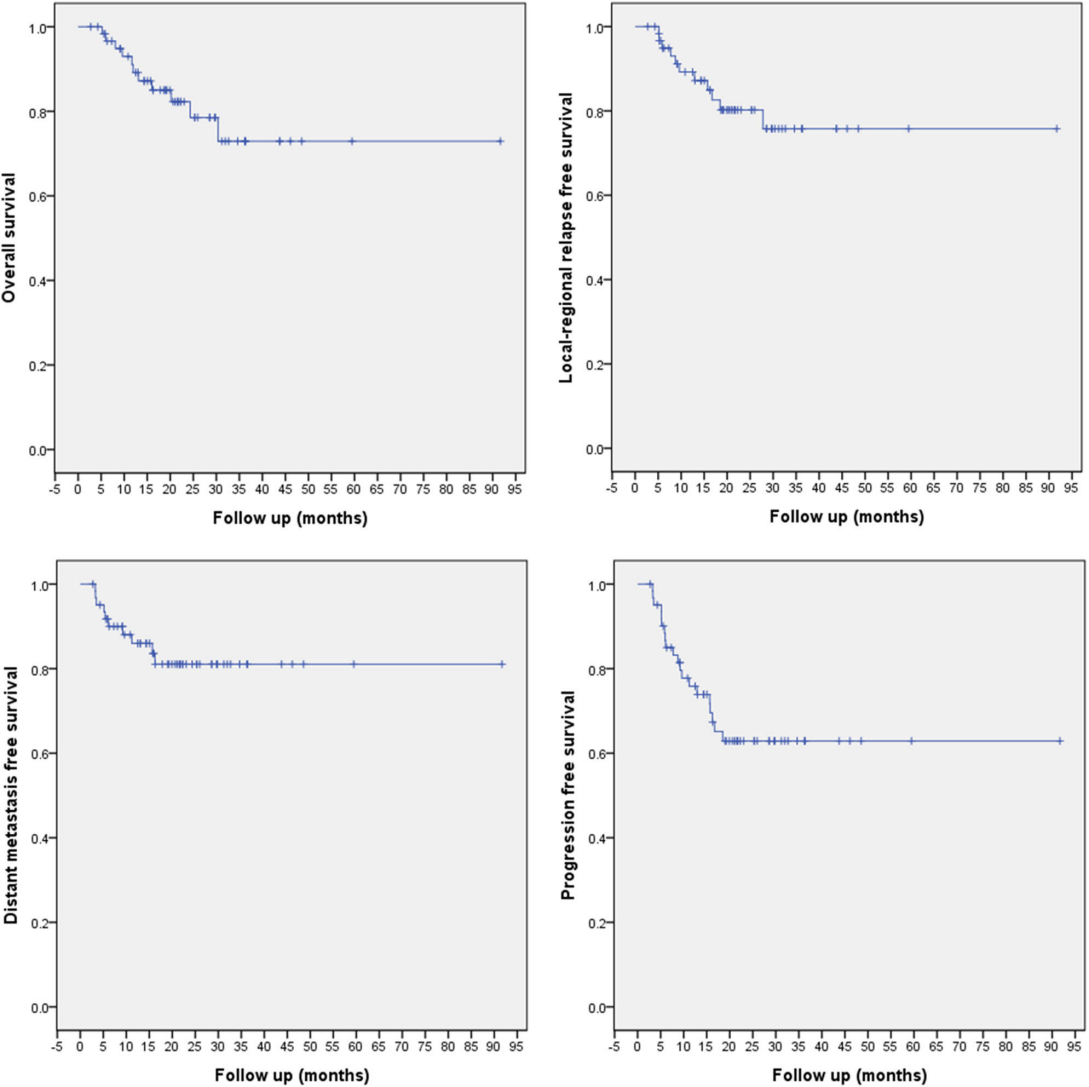
OS, LRFS, DMFS, and PFS of the entire cohort of 62 SBS patients treated with PBRT.

All patients were followed up according to the planned schedule, and the median follow-up time was 20.4 (range 2.73–91.67) months. Eleven patients had deceased. Eleven and 10 events of locoregional recurrence and distant metastasis had occurred, which are detailed later. The 1/2-year overall survival (OS), LRFS, DMFS, and PFS rates for the entire cohort were 91.2/80.2, 89.2/80.2, 86.0/81.1, and 75.8/62.9%, respectively (Figure 2).

#### RT-Naïve Patients

**Figure 3:**
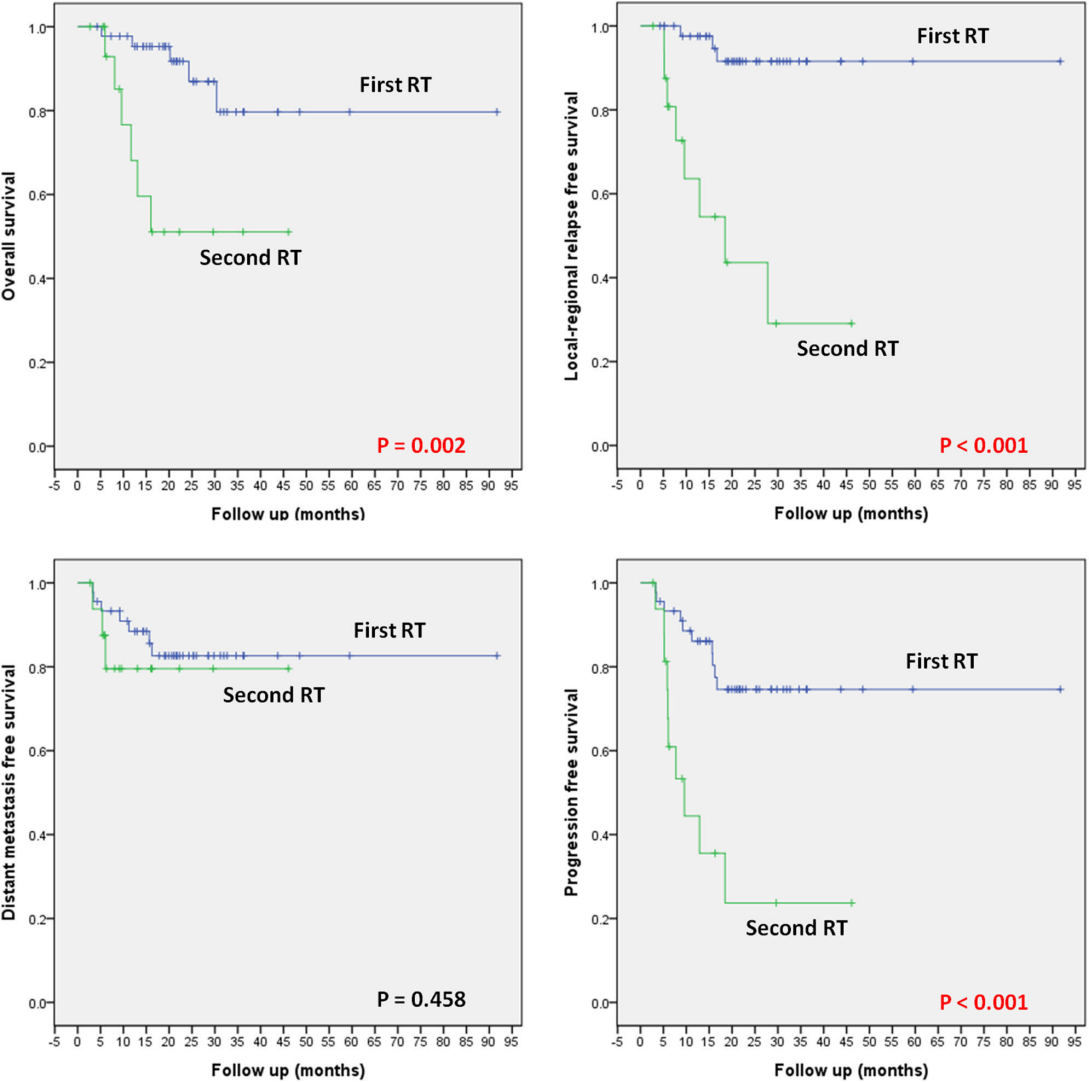
OS, LRFS, DMFS, and PFS according to the status of prior RT (i.e., first-time RT vs. re-irradiation).

Among the 45 RT-naïve patients in this cohort, 5 deceased at the time of this analysis due to distant metastasis (4 cases) or local recurrence (1 case). Locoregional or distant recurrences occurred in 2 and 6 patients, respectively. One additional patient experienced both local and distant recurrence. The 1/2-year OS, LRFS, DMFS, and PFS rates were 95.3/91.8, 97.6/91.6, 88.4/82.6, and 86.1/74.6%, respectively (Figure 3).

#### Re-irradiation Patients

Seventeen patients received re-irradiation using CIRT only. Six of the 17 patients deceased due to local recurrence (5 cases) or massive hemorrhage (1 case). Locoregional or distant recurrences occurred in 6 and 1 patients, respectively. Two additional patients had both local and distant recurrence. The 1/2-year OS, LRFS, DMFS, and PFS rates were 68.1/51.1, 63.6/43.6, 79.5/79.5, and 44.4/23.7%, respectively (Figure 3).

### Adverse Effects

Grade 1/2 oral mucositis and dermatitis of radiation area were the most commonly observed acute adverse effects (17.7/6.5 and 17.7/1.6%, respectively). Grade 3 mucositis was observed in only 1 patient. Another patient with previous radical radiotherapy experienced grade 4 hemorrhage during the treatment and immediately received embolization of the bleeding artery then completed the planned CIRT for radiation-induced second primary pleomorphic sarcoma. The same patient died from hemorrhage (grade 5) at 3.4 months after the completion of PBRT. No other ≧ grade 3 acute radiation-induced toxicity (Tables 2, 3).

**Table 2:** Types and frequency of acute toxicities using CTC.AE.

**Toxicity**	**Grade**									
	**1**		**2**		**3**		**4**		**5**	
	**No**.	**%**	**No**.	**%**	**No**.	**%**	**No**.	**%**	**No**.	**%**
Mucositis/mucosal necrosis	11	17.7	4	6.5	1	1.6	1	1.6	0	
Skin	11	17.7	1	1.6	0		0		0	
Pain	0		1	1.6	0		0		0	
Tinnitus	1	1.6	0		0		0		0	

*PBRT was also well-tolerated in terms of late adverse effects. Only grade 1 and grade 2 toxicities were observed except for the same patient mentioned with grade 4 acute toxicity died of severe hemorrhage (grade 5)*.

**Table 3:** Types and frequency of late toxicities using CTC.AE.

**Toxicity**	**Grade**									
	**1**		**2**		**3**		**4**		**5**	
	**No**.	**%**	**No**.	**%**	**No**.	**%**	**No**.	**%**	**No**.	**%**
Salivary glands (dry mouth)	2	3.2	5	8.1	0		0		0	
Decreased hearing	3	4.8	2	3.2	0		0		0	
Skin	3	4.8	0		0		0		0	
Headache	3	4.8	0		0		0		0	
Parageusia	2	3.2	0		0		0		0	
Radiation encephalopathy	2	3.2	0		0		0		0	
Decreased vision	0		2	3.2	0		0		0	
Tinnitus	1	1.6	0		0		0		0	
Posterior cranial nerves damage	0		1	1.6	0		0		0	
Diplopia	0		1	1.6	0		0		0	
Ptosis	0		1	1.6	0		0		0	
Hemorrhage	0		0		0		0		1	1.6

### Prognostic Factors

Univariate analyses (UVA), including sex, age, status of prior RT (RT-naïve vs. re-RT), pathology (non-chondrosarcoma vs. chondrosarcoma), T-category, surgery status, volume of GTV, PBRT type, and total dose, were compared by log-rank test to demonstrate the differences of the survival probabilities of OS, PFS, LRFS, and DMFS, respectively. The results of UVA are detailed in Table 4.

**Table 4:** Univariate analyses for survival outcomes of 62 cases by Kaplan–Meier method (log-rank).

**Variables**	**OS**	**PFS**	**LRFS**	**DMFS**
Sex (male vs. female)	0.155	0.497	0.477	0.802
Age (< vs. ≥ 40)	*0.004*	0.050	0.052	0.980
Re-irradiation for recurrence or second primary sarcomas (no vs. yes)	*0.002*	* <0.001*	* <0.001*	0.458
Pathology (non-chondrosarcoma vs. chondrosarcoma)	0.084	*0.003*	*0.020*	0.170
T-category (T1/2 vs. T3/4)	*0.025*	*0.001*	*0.024*	0.129
Surgery (R0/R1 vs. biopsy/R2)	0.166	*0.061*	0.148	0.217
GTV (< vs. ≥ 47.45 cm^3^) median	0.169	*0.011*	*0.041*	0.102
PBRT type (IMPT vs. CIRT vs. IMPT + CIRT)	0.624	0.351	0.338	0.640
Total dose (≤ vs. > 63 Gy) median	0.197	0.099	*0.042*	0.646

*OS, overall survival; PFS, progress-free survival; LRFS, local recurrence free survival; DMFS, distant metastasis free survival; PBRT, particle beam radiation therapy. The italic values indicates p > 0.05*.

All the factors of UVA were performed in multivariate analyses (MVA) using Cox regression for OS, PFS, and LRFS. Lack of prior RT (i.e., RT-naïve) was statistically associated with robust OS, PFS, and LRFS (*p* = 0.020, 0.010, and < 0.001, respectively) advantages over re-irradiation, with the hazard ratios of 4.66 (1.268–17.120), 3.416 (1.347–8.664), and 11.990 (3.152–45.610), respectively, making it an independent prognostic factor for OS, PFS, and LRFS. Additionally, age under 40 years was associated with improved OS (*p* = 0.001). Early T-disease (T1/2) showed a significant association with better PFS (*p* = 0.024).

## Discussion

Bone and soft-tissue SBS are rare. No randomized evidence has validated the optimal management of SBS. The anatomic constraints from adjacent critical OARs pose challenges to both surgical and photon radiation-based approaches. Also, most subtypes of SBS are relatively resistant to photon-based radiotherapy. In theory, disease control would be improved with escalation of radiation dose. Also, the prevailing use of IMRT, which allows for improved dose distributions, may benefit the therapeutic ratio for malignancies of the base of the skull (19–21). Nevertheless, it is debatable whether such improvement in conformality actually translates into improved outcomes for SBS: with conventional radiation, local control rates have been commonly estimated between 50 and 60% (3 years), with or without surgery, highlighting the difficulty of achieving local disease control with radiation alone (7, 22).

SBS seems to be an ideal indication to be managed by PBRT, considering both physical, and biological advantages. Minimal exit dose (after the Bragg's peak) and substantially reduced penumbra over photon techniques, including IMRT, provides sharper dose gradients between target volume(s) and the critical OARs that constrains the radiation doses. Also, particle beams with higher linear energy transfer (e.g., carbon ion) produces significantly higher biological effectiveness as compared with photon beams (23), a clear advantage for radioresistant histologies such as most subtypes of sarcomas. Clinical data from several retrospective studies showed that PBRT could achieve favorable disease control for head and neck sarcomas or base of skull tumors, including chordoma and chondrosarcoma, even in patients with unresected or recurrent diseases (24–29). In our previous studies, PBRT for head and neck sarcomas, including those involving skull base, produced effective tumor controls, and overall survivals in both primary and recurrent patients; the 1/2-year OS and LRFS for the entire cohort were 92.9/90.0 and 88.4/78.9%, respectively (24, 25). For chondrosarcoma of the skull base, PBRT attained more favorable outcomes; results from Heidelberg Ion Beam Therapy Center reported both the 5-year OS, and local controls were over 90% and over 85%, respectively (26, 28). Most of the other papers included substantial cases of chordoma of the cervical spine or skull base, a condition that has a significantly different biological behavior from bone and soft-tissue sarcomas (18, 26, 27).

Also, PBRT has been successfully used in other malignancies other than sarcomas of the skull base, including those with extensive involvement of the orbits. In a retrospective study of 57 patients with skull base tumors treated with proton therapy or CIRT between 2003 and 2009, a 3-year OS and LPFS rates of 61 and 56% were reported, respectively (30). Results of PBRT for patients with orbital tumors after eye-sparing surgery produced excellent local controls; our center (SPHIC) reported the 2-year OS and LRFS of 100 and 93.3%, respectively, similar to those in MD Anderson Cancer Center of 100 and 100%, respectively (31, 32). However, the literature on the use of PBRT for the treatment of base of skull sarcomas other than chordoma, which are usually more aggressive and challenging conditions that precludes *en bloc* surgical resection due to their anatomical location, is scarce. The 2-year OS and LPFS rates of our patients treated mostly with CIRT were both 80.2%, and those rates of radiation-naïve patients were both > 91.0%. However, direct comparison between the results in terms of survival and disease control of our patient and previous publications may not be meaningful, as pathologies of patients, treatment modalities, and follow-up time differed substantially.

One of the major clinical advantages of PBRT is its favorable profile of treatment-associated toxicity as compared with conventional radiotherapy. In the current cohort, only one patient experienced grade 3 mucositis. Another developed grade 4 mucosal necrosis with bleeding and later died of hemorrhage of the internal carotid artery. Therefore, both the acute and late toxicities observed in our patients were minimal in severity and frequency. Similar findings were observed after PBRT for skull base malignancies using proton therapy and CIRT (30, 33). Overall, historical data showed that moderate to severe (grade 2–5) acute and late toxicities ranged between 10 and 20% for skull base tumors treated with PBRT. With the use of more modern PBRT technology, such as PBS, improved acute, and late toxicities could be further achieved.

Not surprisingly, RT-naïve patients had significantly better OS, PFS, and LRFS (*p* = 0.020, 0.010, and < 0.001, respectively) over re-irradiation. Also, early T-disease (T1/2) showed a significant association with better PFS (*p* = 0.024). These findings were similar to those reported previously for chondrosarcoma or other based on skull tumors treated using PBRT (33). Interestingly, we did not find that the extent of surgery (R0/R1 vs. R2/biopsy) before PBRT improved patients' survival or disease control in MVA, although a trend favoring complete surgery was observed in UVA. However, such findings might be caused by the limited number of R0/R1 patients in our cohort, and many patients who had biopsy were re-irradiated.

Our study also has few important limitations. The foremost is its retrospective nature and the inclusion of a heterogeneous group of patients. However, for a relatively rare condition such as SBS, it will be difficult if not improbable to perform a prospective randomized trial for each histological subtype of the disease. As such, most of the previously published studies provided results of retrospective analyses that included a heterogeneity group of histologic subtypes, and many included chordoma as well. Second, partly due to the heterogeneity in their histological diagnosis, patients' treatment in terms of surgery and chemotherapy varied substantially. Finally, local recurrence of head neck sarcoma usually occurs within the first 2 years after the completion of radiotherapy (7, 25); thus, our follow-up period of 20.4 months provided an acceptable estimation of patients' survival and disease control. Nevertheless, PBRT-induced long-term toxicities, particularly the CNS structures adjacent to the base skull, would require longer follow-up time.

## Conclusion

Our results showed that PBRT is a promising modality for definitive treatment for patients with base of skull bone and soft-tissue sarcomas, with a high probability of local disease control than historical data and mild to moderate acute and late toxicity. Before, radiotherapy was the most important negative prognosticator of LPFS, PFS, and OS. Also, advanced age and T-disease were significantly associated with poor OS and PFS, respectively. PBRT has the potential to improve the therapeutic ratio, thereby treatment outcomes. The use of PBRT as a definitive treatment modality in skull base sarcomas is worth further investigation, preferably in a prospective fashion, and validation with longer follow-up.

## Data Availability Statement

All datasets presented in this study are included in the article.

## Ethics Statement

This is a retrospective study and was approved by the Institutional Review Board (IRB) of SPHIC.

## Author Contributions

JL: conception and design. LK: administrative support. JL and LK: provision of study patients. JY, XG, JG, JH, WH, XQ, QH, and WZ: collection and assembly of data. JY: data analysis and interpretation. JL and JY: manuscript writing. All authors: final approval of manuscript.

## Conflict of Interest

The authors declare that the research was conducted in the absence of any commercial or financial relationships that could be construed as a potential conflict of interest.
